# A Social Diffusion Model with an Application on Election Simulation

**DOI:** 10.1155/2014/180590

**Published:** 2014-06-05

**Authors:** Jing-Kai Lou, Fu-Min Wang, Chin-Hua Tsai, San-Chuan Hung, Perng-Hwa Kung, Shou-De Lin, Kuan-Ta Chen, Chin-Laung Lei

**Affiliations:** ^1^Institute of information Science, Academia Sinica, 128 Academia Road, Section 2, Nankang, Taipei 115, Taiwan; ^2^Department of Electrical Engineering, National Taiwan University, No. 1, Section 4, Roosevelt Road, Taipei 10617, Taiwan; ^3^Department of Computer Science and Information Engineering, National Taiwan University, No. 1, Section 4, Roosevelt Road, Taipei 10617, Taiwan

## Abstract

Issues about opinion diffusion have been studied for decades. It has so far no empirical approach to model the interflow and formation of crowd's opinion in elections due to two reasons. First, unlike the spread of information or flu, individuals have their intrinsic attitudes to election candidates in advance. Second, opinions are generally simply assumed as single values in most diffusion models. However, in this case, an opinion should represent preference toward multiple candidates. Previously done models thus may not intuitively interpret such scenario. This work is to design a diffusion model which is capable of managing the aforementioned scenario. To demonstrate the usefulness of our model, we simulate the diffusion on the network built based on a publicly available bibliography dataset. We compare the proposed model with other well-known models such as independent cascade. It turns out that our model consistently outperforms other models. We additionally investigate electoral issues with our model simulator.

## 1. Introduction

Huge success of viral marketing nowadays clearly shows that acquaintances indeed greatly influence people adopting a new or different opinion. This implicates that people, in a way, attempt to plant their intrinsic ideas, opinions, or preferences in others' minds through exchanging opinions over and over in different circumstances. One interesting and long-discussed scenario is election. Elections in the modern world are an essential mechanism to aggregate the opinions of the masses and to make joint decisions for a variety of purposes. People share thoughts and even attempt to convince others to adopt their attitudes during the election season.

As social media such as Facebook are widely utilized, it becomes quite convenient for people to manifest themselves. Social media exposure grants people a hitherto wide range to deliver their views. Social media extremely accelerates and facilitates such opinion-exchange interactions among individuals. As opinions interflow, the intrinsic opinions of an irresolute person could eventually be assimilated to those of the determined ones. Then a consensus or a public opinion appears.

From a research aspect, understanding the progress of human negotiation benefits the real world applications. For instance, social scientists would wonder to what extent the opinions' exchange among friends can affect each other's viewpoints. Campaign companies would inquire how to promote a candidate with limited budgets. Such questions are not easy to answer via a user study, particularly when the number of participants becomes huge.

Opinion diffusion on social networks has been studied for decades. Unfortunately, many previous models, such as the Independent Cascade Model, Linear Threshold Model, SIR/SIS model, and heat diffusion model, cannot manage the election scenario intuitively due to the following two reasons. First, people have their intrinsic opinions more or less, which is absent in the aforementioned models. In such a manner, people may not serve as neutral relays. People amplify the opinions they stand for and deamplify opinions they stand against. Second, opinions could be multidimensional, for example, a viewpoint for multiple electoral candidates. Most diffusion models adopt single values to represent the opinions for simplicity. A single value clearly cannot directly represent the views about multiple candidates. Our goal is to design a suitable diffusion model which is capable of managing the propagation of viewpoints. Up to date we have not yet seen too many computational approaches with systematic and quantifiable studies on this issue.

Inspired by the real world phenomena, we have realized several preferable properties to manage the information diffusion like opinion polling. The properties are* high-dimensional media*,* input dependence*,* deterministic convergence*, and* consensus*. A summary of the properties is as follows. First, we prefer the media (which represents preference toward candidates) propagated throughout the process being a unit vector because, democratically, individuals (or nodes) have equal rights in casting votes. Second, the preference distribution should be significantly affected by the initial intrinsic preference as well as the neighbors through social network. Finally, we hope the propagation converges eventually, and a common trend appears after numerous interactions [1]. In this paper, we show that our model is the only one satisfying all properties among the existing models.

The novelty and contributions of this paper can be viewed from several different angles.

1. This work strategically demonstrates a plausible process to answer a set of real world problems.
  - We start by designing a preference negotiation model (with theoretical guarantees) to manage high-dimensional information. We assess the quality of this model by proving its convergence and several other important properties.
  - We conduct an experiment to demonstrate the validity of our model in predicting the change of citation preference among authors through collaboration networks.
  - To build the diffusion simulation framework, we further devise suite of satellite algorithms for preference profile sampling, deployment, and seed-voter selection.
2. Our case studies, in practice, have provided the concrete solutions to the real world problems of concern.
  - The simulation shows that election outcomes can be significantly affected by the social factor. We find that each individual preference profile extremely changes after a preference diffusion. Using Kendal *τ* coefficient to measure the similarity before and after the diffusion, it usually results in a coefficient below 0.5.
  - With the simulation, we additionally examine several well-known voting schemas to verify their vulnerability to vote-buying. Among them, Borda Count voting schema performs best to resist vote-buying. Plurality voting is the most vulnerable to manipulation.

## 2. Preliminary

We review the previously done works related to the information diffusion and electoral issues.

### 2.1. Diffusion Model

To develop models for diffusion simulation or prediction, researchers unearth the underlying mechanisms or the inherent patterns of information diffusion from real word phenomena and utilize these findings.

The Linear Threshold Model (LT model for short) [2, 3] and Independent Cascade Model (IC model for short) [3, 4] are the most well-known and fundamental ones to describe how the information propagates step by step in a network. Inspired by the ideas of the two models, various models have been proposed later for more specific scenarios.

The LT model at first intends to describe the process of shutdowns due to chain effect of energy overload in a power grid. The concept is then adopted for simulating the information diffusion. In the LT model, nodes in a network are the containers of energy (information) and the amounts carried are represented as real values. Each node has a predesignated carrying capacity and initially carries no energy. Once the simulation proceeds, some nodes are assigned as the early adopters, the first groups gaining energy (information), to carry energy, and the carried amount increases progressively. Once the amount of carried energy exceeds their capacity, the nodes become active (overload) and pass excessive energy to other linked nodes. This leads to a propagation of power overload.

With an operation similar to the LT model, the IC model further simplifies the carried information as a binary value. Nodes become active once they receive the information passed from the linked neighbors. There is no predetermined capacity for nodes in IC model. Instead, each edge is associated with a real number representing the probability of successful information pass along it. During the diffusion, the active nodes continually attempt to send out the information through edges until all linked neighbors become active.

Kempe et al. (2003) [3] generalized the IC model by introducing a General Cascade Model. Gruhl et al. (2004) [5] and Leskovec et al. (2006) [6] proposed generative model to simulate blog essay generation based on the IC Model. These models assume nodes can turn from inactive to active given a certain probability for cascading. Based on the LT model and the IC model, Saito et al. (2010) [7] proposed the Asynchronous Linear Threshold Model and Asynchronous Independent Cascade Model.

Another influential line of research, following the success of the PageRank algorithm, puts the propagation process in an explicit recursive mathematical form. Heat diffusion [8, 9] is a physics phenomenon describing heat flows from high temperature positions to low temperature positions. Inspired by the heat diffusion, Ma et al. (2008) [8] proposed a model to analyze candidate selection strategies for market promotion. The process is formulated as

(1) $$\begin{array}{c} \frac{f_{i}\left(t+\Delta t\right)-f_{i}\left(t\right)}{\Delta t}=\alpha \underset{j:(v_{j},v_{i})\in E}{\sum }\left(f_{j}\left(t\right)-f_{i}\left(t\right)\right), \end{array}$$

where *f*_*i*_(*t*) is the heat of node *i* at time *t* and *α* is the thermal conductivity, namely, the heat diffusion coefficient.

In heat diffusion process, each vertex receives heat from its neighbors, which is similar to our model. The major difference, which will be discussed in detail in the following section, is that heat diffusion model lacks a normalization phase (since it considers only the propagation of one value) and a fusion phase (because the heat itself can disappear after diffusion, so there is no need to fuse on heat diffusion model).

Inspired by these previous works, our model takes the strong points of these approaches, namely, their focus on mimicking social interaction traits such as forming consensus as well as their incorporation of structural information into the propagation process, and blends them into a more coherent framework that could be used to answer real world problems mentioned in the introduction.

### 2.2. Electoral Issues

In 1992, Bartholdi III et al. [10] first studied the complexity of the process to determine needed actions by organizer to add or remove candidates to manipulate election results (where it is recognized as the classical social choice theory). However, they did not propose any model for the interactions between voters. Gibbard [11] and Satterthwaite [12] showed that every election scheme with at least three possible outcomes is subject to individual manipulation. This means the minority has a chance to manipulate the group decision to secure a preferred outcome. Gibbard and Satterthwaite also addressed the computational difficulty in minority manipulation. However, their model assumes the independence of voters, which does not consider nor discuss the effect from other voters on voter's preference. Existing studies in this direction mainly focus on the complexity and feasibility issues, which is very different from our goal.

Liu (2009) [13] attempted to check whether the preference distribution changes if the number of political experts in a communication network increases. They use an agent-based model for simulation. Each agent in the model maintains a binary value toward a candidate (instead of a real value or ranking) and simply disseminates the values to other agents in the nearby 3 by 3 matrix.

Yoo et al. (2009) [14] proposed semisupervised importance propagation model. Their idea is, to some extent, similar to our “fusion phase” by adding the original score into the accumulated score obtained from the neighbor. The difference between their model and ours is that theirs deals with a single value instead of a vector, and therefore they do not perform the normalization over candidate scores like we do.

The election manipulation is a long-discussed issue. Nevertheless, the social factor is absent in these works. Here we bring a marriage between the social network analysis and the electoral issues.

## 3. The Proposed Model

We here propose the diffusion model to unearth how the communications affect the individual decisions. Abbreviations section lists the notations used in the rest of the paper.

### 3.1. Preference Propagation Model

We first define a preference profile *p*_*v*_ of an individual *v*, which is a *k*-dimensional vector that represents *v*'s preference toward *k* different candidates. The *j*th element in *p*_*v*_ is an integer in [1, *k*] indicating this individual's preference for candidate *j* (smaller numbers denote higher ranks). To facilitate the operation of the preference profiles, we translate *p*_*v*_ into a score vector *s*_*v*_, for all *v*, using the following equation:

(2) $$\begin{array}{c} s_{v}\left[i\right]=\frac{\left(k-p_{v}\left[i\right]+1\right)}{T},\;\forall i\in 1,2,\ldots ,k, \end{array}$$

where *T* = *k*(*k* + 1)/2. This transformation can be regarded as a normalization process as in *s*_*v*_ not only does the preferred candidate receive higher score but also the sum of all elements equals 1. Using the score vector of each individual, we can create an *n* by *k* matrix *S* = (*s*_*v*_1__, *s*_*v*_1__,…, *s*_*v*_*n*__)^*t*^ denoted by the preference matrix. We denote the preference matrix of a given time stamp *t* since the propagation process starts as *S*(*t*).

The information propagates one iteration after the other in our model, and each iteration consists of three phases:* propagation*,* normalization*, and* fusion*.

In the propagation phase, each node *v* synchronically propagates the preference score vector *s*_*v*_ to the neighboring nodes. To describe such operation mathematically, we define an *n* × *n* forward transition matrix *F* such that the multiplication of *F* and *S*(*t*) represents the score of each node obtained from all neighbors after this phase. We denoted it by *S*_*p*_(*t*).

We assume the edge directions in a network *G* reveal the direction of influence. Therefore, *F* = (*KA*)^*t*^, where *K* is a diagonal matrix with the inverse of degree of each node in the diagonal and *A* is the adjacency matrix of *G*. Note that *F* is identical to the forwarding matrix of a random walk algorithm. The only difference is that *F* in a random walk algorithm is multiplied by a vector instead of by a matrix *S*.

In *S*_*p*_, each row represents the neighbors' accumulated preference scores toward each candidate. Unlike *S*, the elements in each row of *S*_*p*_ do not add up to one. To ensure that every individual has equal influence while casting votes, we normalize each row of *S*_*p*_ so that its elements add up to one. Therefore, in the second phase, *S*_*p*_ is multiplied by an *n* × *n* diagonal normalization matrix *N*, where each element in the diagonal of *N* is equal to the sum of all elements in the corresponding row of *S*_*p*_. After the second phase, we will obtain a new scoring matrix *S*_*n*_(*t*) = *NFS*(*t*).

The major difference between our propagation model and the diffusion models for electricity/heat (see Section 2 for more details) lies in the intrinsic difference of the media that are propagated. Electricity or heat flows from one place to another (that is, a flow from node *A* to node *B* implies the material does not exist in *A* anymore). Opinions, by contrast, do not vanish after propagation (that is, *A*'s inclination towards a candidate does not disappear even after bringing his or her opinions to *B*). Therefore we add a third phase to include a fusion model that integrates an individual's own opinions *S*(*t*) with the opinion *S*_*n*_(*t*) gathered from its neighbors.

In the fusion phase, we introduce a parameter for each individual: the susceptible ratio, a real number *ϵ* ∈ [0,1] that represents how easily an individual can be affected by others. Given a susceptibility parameter for each individual, we can then create a susceptible matrix *E*, an *n* × *n* diagonal matrix with the *ϵ* value of each individual in the diagonal. If *E* is equal to the identity matrix *I*, which would imply all individuals are equally and highly susceptible to one another, then *S*(*t* + 1) should be equivalent to its neighbors' opinion *S*_*n*_(*t*). On the opposite side, if *E* is equal to the zero matrix, implying all individuals are impervious to one another, then *S*(*t* + 1) should be identical to *S*(*t*). Thus, after one iteration of propagation, the preference score matrix can be represented as

(3) $$\begin{array}{c} S\left(t+1\right)=\left(I-E\right)S\left(t\right)+ENFS\left(t\right)=\left(\left(I-E\right)+ENF\right)S\left(t\right). \end{array}$$

Note that we assume that *E* does not change over time, and neither does *F* (which is only dependent upon topology). Interestingly, at first glance one might assume that *N* changes iteratively; it actually does not. Because the sum of each column in *F* equals 1 and the scores are always normalized for all candidates, it is not hard to prove that

(4) $$\begin{array}{c} N_{ij}=\{\begin{array}{cc} {\left(\overset{n}{\underset{j=1}{\sum }}F_{i,j}\right)}^{-1} & \text{when}\;i=j\\ 0 & \text{otherwise}, \end{array} \end{array}$$

which depends only on *F*. Therefore, we can rewrite *S*(*t* + 1) as *XS*(*t*), where *X* is a time-independent matrix, which becomes an important feature for the proof of convergence in the next section.

The above concludes one iteration of propagation. In the next iteration, *S*(*t* + 1) becomes the initial preference score for the individuals and the same process can be executed to obtain another round of propagation results *S*(*t* + 2).  Algorithm 1 is the algorithm for our model.

### 3.2. Proof of Convergence and Consensus

In this section, we show the convergent property of our proposed scheme. The score matrix becomes invariant after a sufficient number of propagations. Moreover, we show that given certain conditions all rows in the converged score matrix are identical. In other words, a consensus within a community will eventually be reached through information propagations in our model.

Let *X* denote the overall preference propagation operation of all three phases explicitly laid down in the previous section,

(5) $$\begin{array}{c} S\left(t+1\right)=XS\left(t\right)=\left[\left(I-E\right)+ENF\right]S\left(t\right). \end{array}$$

To provide intuition for the forthcoming deductions and to borrow results of the properties of *X* from Section 3.1, we start by pointing out the similarities as well as the differences between *X* and the PageRank matrix *G*. First, the entity *X* acting on *S*(*t*) is actually a matrix consisting of the vectors of probabilities instead of a simple vector of probabilities. As a result, the columns of *X* do not add up to 1 (only the rows do) and therefore it is not a stochastic matrix. Furthermore, a social personal relationship network is intrinsically more localized compared to the World Wide Web, and, as such, the favorable positive definite property enjoyed by *G* does not necessarily hold for *S*. That said, these complexities, while no doubt complicating the theoretical treatment of our algorithm, are in fact a natural manifestation of the increased richness of our target of research in hand—social networks.

We start our deduction of the convergence of *X* by enlisting the Perron-Frobenius theorem [15] which states that an irreducible, acyclic matrix has a single eigenvalue that is strictly larger than the others. Under the assumption that the graph being induced by *X*, *G*_*X*_, is strongly connected and that the weights matrix *E* has entries smaller than one but not all zeros, *X* is irreducible and acyclic and thus applies to the Perron-Frobenius theorem. We denote the dominant real positive eigenvalue of *X* by *r*. Armed with this fact, we are able to transform *X* into its Jordan canonical form

(6) $$\begin{array}{c} X=P^{-1}J_{X}P,\;J_{X}=\left(\begin{array}{ccc} J_{X_{1}} & 0 & \ldots \\ 0 & J_{X_{2}} & \ldots \\ \vdots & \vdots & \ddots \end{array}\right), \end{array}$$

by which the leading block *J*_*X*_1__ is a 1 × 1 matrix [*r*], and other *J*_*X*_*i*__'s correspond to their strictly smaller eigenvalues *λ*_*X*_*i*__. Since, by the rules of matrix multiplication, the effects of *X* on *S*(*t*) can be analyzed one by one with respect to *S*(*t*)'s column vectors without loss of generality, we will proceed with our proof of *S*(*t*)'s convergence by concentrating on *S*(*t*)'s column vectors which we denote by lower case *s*(*t*). Decomposing *s*(0) into the sum of *X*'s eigenvectors, *c*_1_ *v*_1_ + *c*_2_ *v*_2_ + ⋯, we obtain the general form of the time evolution of *s*(*t*),

(7) $$\begin{array}{c} s\left(t\right)=J_{X}^{t}\left(c_{1}v_{1}+c_{2}v_{2}+\cdots \right)=r^{t}\left(c_{1}v_{1}+b_{t}\right), \end{array}$$

where

(8) ||bt||=1rt||JX2tc2v2+⋯||≤∑i=2|V|(|λxi|r)t||civi||⟶0, as  t⟶∞.

The above shows that ||*b*_*t*_|| converges to zero when *t* is large, and therefore *S*(*t*) converges to *r* ^*t*^(*c*_1_ *v*_1_). To get an intuition for the speed of this convergence, we turn to a special case where the susceptible ratios are identical; that is, *E* is a scalar *ϵ*. In this case, we apply the Perron-Frobenius theorem again on *NF* and we again obtain *NF*'s Jordan form

(9) $$\begin{array}{c} NF=P^{-1}J_{NF}P,\;J_{NF}=\left(\begin{array}{ccc} J_{NF_{1}} & 0 & \ldots \\ 0 & J_{NF_{2}} & \ldots \\ \vdots & \vdots & \ddots \end{array}\right). \end{array}$$

However, note that since it needs not to be acyclic, it is strictly larger than the other. Now, using this basis we find that *X* is equal to

(10) $$\begin{array}{c} \epsilon \left(\begin{array}{ccccc} \ddots & \ddots & & & 0\\ & \frac{\left(1-\epsilon +\epsilon \lambda_{NF_{i}}\right)}{\epsilon } & 1 & & \\ & & \frac{\left(1-\epsilon +\epsilon \lambda_{NF_{i}}\right)}{\epsilon } & 1 & \\ & & & \frac{\left(1-\epsilon +\epsilon \lambda_{NF_{i}}\right)}{\epsilon } & \ddots \\ 0 & & & & \ddots \end{array}\right). \end{array}$$

Since a Jordan canonical form is unique, we obtain *λ*_*X*_*i*__ = (1 − *ϵ* + *ϵλ*_*NF*_*i*__)/*ϵ*. From this result, we confirm that when *ϵ* = 0, *X* degenerates to the trivial diagonal case and that as *ϵ* approaches 1, the rate of convergence is geometrically proportional to *ϵ*/*r*.

We are now one step away from the final proof of *S*'s convergence. Recalling that *s*(*t*) → *r* ^*t*^ *c*_1_ *v*_1_, once *r* ≤ 1 is established, *S*(*t*) converges. To prove this, we take advantage of the Collatz-Wielandt theorem which gives the following formula for *r*: *r* = max⁡_*x*∈*N*_ *f*(*x*), where *f*(*x*) = min⁡_1≤*i*≤*n*;*x*_*i*_≠0_[*Xx*]/*x*_*i*_ and *N* = {*x* | *x* ≥ 0  with  *x* ≠ 0}.

We begin by asserting that the upper bound of *f*(*x*) is 1. To prove this, we suppose the opposite holds, that means there exists *x* such that *f*(*x*) = min⁡_1≤*i*≤*n*;*x*_*i*_≠0_[*Xx*]/*x*_*i*_ = *α* > 1. This implies the following list of equations:

(11) 1<α≤1x1(X11x1+X12x2+⋯+X1nxn)⋮1<α≤1xn(Xn1x1+Xn2x2+⋯+Xnnxn).

Note that ∑_*j*=1_^*n*^ *X*_*ij*_ = 1, ∀*i*. Thus, the above list of equations can be arranged into

(12) X12(x2x1−1)+X13(x3x1−1)+⋯+X1n(xnx1−1) >0   ⋮Xn1(x1xn−1)+Xn2(x2xn−1)+⋯+Xn(n−1)(xn−1xn−1) >0.

However, by denoting *i* by the subscript that has *x*_*i*_ = max⁡_1≤*j*≤*n*_ *x*_*j*_ and remembering that *X* is a nonnegative matrix, one of the above equations would not hold:

(13) $$\begin{array}{c} X_{i1}\left(\frac{x_{1}}{x_{i}}-1\right)+X_{i1}\left(\frac{x_{2}}{x_{i}}-1\right)+\cdots +X_{in}\left(\frac{x_{n}}{x_{i}}-1\right)>0. \end{array}$$

This justifies the assertion that *f*(*x*) ≤ 1. Combining this result with the observation that the trivial vector (1,1,…) yields *f*(*x*) = 1, we conclude that max⁡_*x*∈*N*_ *f*(*x*) = 1. Therefore, *r* = 1, and *S*(*t*) converges to *c*_1_ *v*_1_.

**Figure 1:**
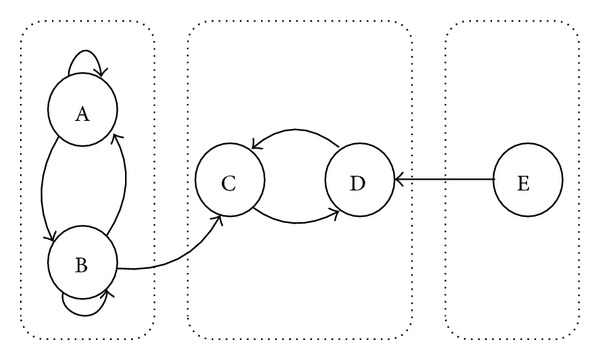
Nodes A, B form an opinion leader SCC, while node C by itself is another opinion leader SCC. Nodes E and D form an opinion follower SCC.

For networks that are not strongly connected we can always find the SCCs in linear time, and the problem reduces to the smaller “source SCCs” of the network since the matrices of all the other SCCs have a Perron root smaller than 1 and their elements eventually vanish. For the remaining source SCCs, since no vertices have susceptibility ratios equal to 1, according to the above results they all converge. The net effect is exemplified by the stark difference between the individuals belonging to the various source SCCs and the rest. Whereas source SCC vertices will converge to their own respective common values, the others may converge to different values and act as followers in terms of aligning their own preferences to the weighted average of those belonging to the sources.  Figure 1 gives an example of such phenomenon. Let the initial preference matrix of all the nodes in Figure 1 be

(14) $$\begin{array}{c} \left(\begin{array}{ccccc} s_{\text{A}} & s_{\text{B}} & s_{\text{C}} & s_{\text{D}} & s_{\text{E}}\\ s_{\text{A}}^{\prime } & s_{\text{B}}^{\prime } & s_{\text{C}}^{\prime } & s_{\text{D}}^{\prime } & s_{\text{E}}^{\prime }\\ s_{\text{A}}^{\prime \prime } & s_{\text{B}}^{\prime \prime } & s_{\text{C}}^{\prime \prime } & s_{\text{D}}^{\prime \prime } & s_{\text{E}}^{\prime \prime } \end{array}\right)\begin{array}{c} \text{candidate}1\\ \text{candidate}2\\ \text{candidate}3, \end{array} \end{array}$$

where each row in the preference matrix denotes each node's preference for candidates 1, 2, and 3, respectively. Then after infinite number of propagations, the preference matrix will become

(15) $$\begin{array}{c} \left(\begin{array}{ccccc} s{\left(\infty \right)}_{\text{AB}} & s{\left(\infty \right)}_{\text{AB}} & s{\left(\infty \right)}_{\text{C}} & s{\left(\infty \right)}_{\text{D}} & s{\left(\infty \right)}_{\text{E}}\\ s{\left(\infty \right)}_{\text{AB}}^{\prime } & s{\left(\infty \right)}_{\text{AB}}^{\prime } & s{\left(\infty \right)}_{\text{C}}^{\prime } & s{\left(\infty \right)}_{\text{D}}^{\prime } & s{\left(\infty \right)}_{\text{E}}^{\prime }\\ s{\left(\infty \right)}_{\text{AB}}^{\prime \prime } & s{\left(\infty \right)}_{\text{AB}}^{\prime \prime } & s{\left(\infty \right)}_{\text{C}}^{\prime \prime } & s{\left(\infty \right)}_{\text{D}}^{\prime \prime } & s{\left(\infty \right)}_{\text{E}}^{\prime \prime } \end{array}\right), \end{array}$$

in which the preferences of nodes A and B in Figure 1 for candidate 1 converge to the common value *s*(*∞*)_AB_, for candidate 2 converge to the common value *s*(*∞*)_AB_′, and for candidate 3 converge to the common value *s*(*∞*)_AB_′′. However, for nodes D and E, given that the SCC composed by them {D, E} is under the influences of both opinion leaders SCC {A, B} and {C}, their eventual preferences instead of aligning themselves to a common value become a linear combination of the preferences of {A, B} and {C}. The exact details of this combination depend on the structure of the network.

The preference propagation model simulates this unique behavior of people by projecting the preferences' vector onto the leading uniform eigenvector denoting equilibrium. In addition, it also attempts to mimic the real world by distinguishing the opinion leaders from the followers. As with its real world counterpart, this process is solely determined by the initial preferences of every individual and the structure of the embedding social network.

**Figure 2:**
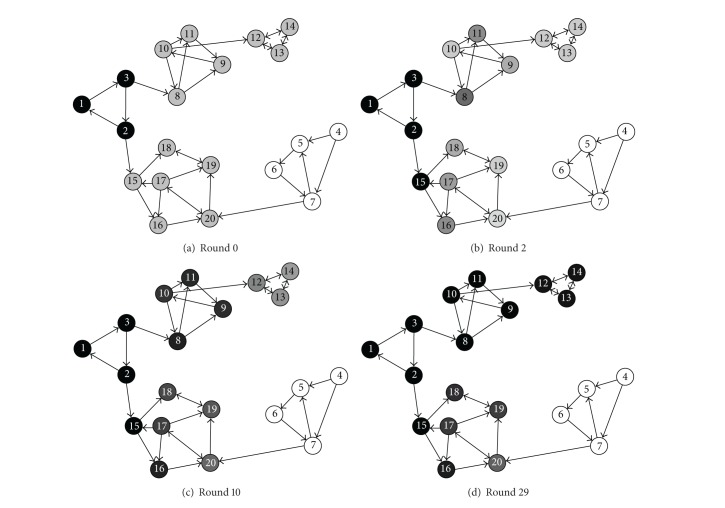
Time evolution of preferences held by nodes in a social network, demonstrating the effects of opinion leaders creating their own consensus and passing it down to opinion followers in a cascading manner.

Another example is shown in Figure 2, time evolution of preferences held by nodes in a social network demonstrating the effects of opinion leaders, creating their own consensus, and passing it down to opinion followers in a cascading manner. We see that the opinion follower SCC composed by nodes 15 to 20 is colored with various shades of gray depending on its distance to the two opinion leader SCCs composed by nodes 1 to 3 and 4 to 7. We observe that the preference of the opinion leader SCCs 1 to 3 is first passed to the opinion follower SCCs 8 to 11 (in 10th propagation round) and then subsequently passed to the opinion follower SCCs 12 to 14 through the efforts of SCCs 8 to 11 in a cascaded manner.

This simple example demonstrates that the strongly connected source components form the opinion leader groups, while each follower node is affected by (i.e., linear combination) the opinions of its surrounding opinion leader groups. Our framework models the real world observation about how less-convinced personnel are affected by the mass opinions they encountered.

### 3.3. Comparison with Other Models

We here discuss what the most salient characteristics of a successful social model are based on common observations and beliefs, in an attempt to contrast the most distinguishing features of our model with the other previously proposed frameworks.

*High Dimension Media*. Since a personal preference describes the order of preference of all possible candidates, the media in an ideal model should be represented as ordered lists instead of as a single value. Most of the propagation models such as Linear Threshold Model, Cascade Independent Model, or DiffusionRank model, unfortunately, only handle binary or real value in propagation.

*Topology Dependence and Input Dependence*. The word of mouth is the main strategy for a person to affect others. The real world process of guiding friends toward the adoption of self-preference goes mutually and simultaneously. To state such phenomenon, the outgoing persuasions of a person should ideally become a combination of self-preference and the incoming preferences. An ideal model should take into account both network structure and initial personal preference. Moreover, we would like a model's way of incorporating these two factors to be as natural as possible, instead of relying on ad hoc stopping designs or simply restricting the number of time nodes or individuals' interactions. 

*Deterministic Convergence*. Of course an ideal model should converge or end eventually, or else it would be difficult for the modeler to interpret the results. As far as we know, there are currently two kinds of designs to achieve such a convergence. The first one, such as LT model and IC model, attaches a binary status to each node in a network to determine whether it is visited. The* inactive* status means the node is not yet visited while the* active* status means the node is visited. With such design, preference propagation to inactive nodes can be easily monitored. Moreover, the propagation converges in such model when none of the existing nodes can change the status anymore.

Following the success of the PageRank algorithm, the second popular approach is building the convergence mechanism into a model inherently, so that after sufficient iterations the model converges and produces a definite result.

To make results easily analyzable, convergent models that can generate identical results, given both the same initial preferences of nodes and network structure, are preferred. 

*Consensus*. The problem of reaching a consensus among agents has been studied since around 1970 [16, 17] with simulation models such as the voter model [1]. Mossel et al. gave a theoretical proof that the consensus could be reached with the voter model [18]. Thus an ideal model should be able to reflect specific common traits. In particular, we observe that one such universal trait is people in the same community (i.e., SCC) having the tendency to align their preference after sufficient exchanges. This translates into the fact that an ideal model should contain some kind of homogeneity inside a group.

To see how our model and other proposed frameworks capture the above characteristics of real world social interactions, we conducted several experiments and recorded their results in Table 1 for ease of comparison. We particularly chose models that are most representative in their own stance, namely, the Linear Threshold Model, Independent Cascade Model, PageRank model, and DiffusionRank model, for comparison. Note that since the propagating media in these models are not a vector of preference, we made the following enhancements for each of them to handle such cases. For the LT and IC models, we assume that each vertex initially held approval for its top *k* preferred candidates (nonapproval for the others), and thus, for every candidate, we can obtain a list of seeds as inputs into the LT and IC models. We then execute the model separately on each candidate, gather their results, and normalize them to form the final preference of each vertex. For the PageRank and DiffusionRank models, given that they can take real values as inputs, we simply executed these models separately for each candidate in the preference list and then integrated the results to be a vector of real numbers.

As shown in Table 1, we see that our model is the only model that operates directly on a list of preferences, whereas other models work restrictively on single boolean or real values and have to be executed separately to obtain a joint preference, which fail to consider the correlation of the preference score among candidates. We note that all models provide convergent results. Besides, since the IC model carries a random component, it does not deliver repeatable final preference results.

To examine whether these models can give a kind of consensus to nodes that belong to a strongly connected network, we execute all models on a strongly connected graph until they naturally stop or converge. It turns out that, except for our model, none showed signs of reaching consensus among the final output preferences. Note that our model does not produce consensus given non-SCC components.

To see whether these models take into account the initial preferences held by nodes, we fed all models with six different initial preferences and see whether they give six different results. It is not surprising that the PageRank model returns identical results regardless of the input, indicating that it takes into account only the structure of the network but ignores the initial preferences held by each node or individual. In conclusion, our model is the only framework that supports all five criteria set by observations from real world social networks.

## 4. Experiment

In this section, we compare our model with well-known diffusion models to evaluate the performance. We examine whether all the aforementioned algorithms, including ours, can capture the preference transition in social networks to a certain extent. To conduct such validation, we require the information such as the network structure and the node preferences over time.

### 4.1. Preference Data

The citations of scientific research papers implicitly reveal the research interests of the authors. In other words, we believe that the acts such as citing or submitting to the journals or the conferences would be an indicator of the authors' interests. By utilizing this fact, we can infer the researchers' preference from their corresponding top frequently cited conferences and journals. We have further realized that one author's preference could be influenced by the other coauthors. It is particularly correct for advisor-student relationship since the advisors and students usually affect each other's research interests. We therefore have designed an experiment to model how researchers' preferences can be affected by the collaborators.

We use KDD Cup 2003 ArXiv HEP-TH (High Energy Physics Theory) citation network [19] with the corresponding paper meta information as our evaluation dataset. This dataset contains the citations from 1992 to 2003. We select the top 16 journals that possess most papers as the candidates to construct the preference lists and construct the yearly preference lists for all authors. A preference list consists of the citation count of the corresponding journals within one year. Thus, for each author, we have 12 lists representing their preferences.

The reason we use the citations rather than the publications of authors is that the publications imply not only preference but also capability. To fairly present the interests, we use the citations. In addition, we construct a collaborative network from this dataset as the underlying social preference diffusion backbone. To easily perceive the changes in interests, we remove the authors who had fewer than 5 publications in the dataset, which results in a network with 2683 nodes.

### 4.2. Model Comparison

Since we already have all the required information including network structure and preference transition, the next step is to study which diffusion model predicts the preference transition better. We assume a good diffusion model could capture the progression of the authors' research interests through collaborations. To do so, we initially set up the node preference according to the actual data in year *x* and then compare the predicting results with the actual preference in year *x* + *k*. The following issues are noted in the experiment.

*High Dimension Media*. To represent the order in preference toward all candidates, the media in an ideal model ought to be an ordered list instead of a single value. Nonetheless, most well-known diffusion models, such as LT, IC, and DiffusionRank, only treat the media as boolean or real number. For comparison, we exploit these models in our problem by executing them independently for each candidate. We evaluate the candidate rank based on each independent diffusion result. 

*Determinism of the Final State*. Except for the IC model, outcome of all the models mentioned above is deterministic. Because the parameter (i.e., diffusion probability) in IC model is a nondeterministic factor, we execute the experiment 20 times and average the results. 

*Initialization*. Because the media in LT and IC models are not native for high dimension, we singly process the propagation for each candidate. That means, in our experiment, the active mode of top 1% authors to a specific publisher is initially set active in LT and IC models, while the rest of publishers are set inactive. We further set the diffusion probability of each edge as 1/*N*, where *N* is the degree of its source node in IC model. In LT model, we assign links with identical weight and nodes with the same threshold. The parameters in LT and IC are then tuned to find the optimal outcome. The propagation process is executed multiple times with different thresholds and the performance is averaged. For DiffusionRank model, we use the parameter settings suggested by the authors of [9].

### 4.3. Experiment Result

Diffusion models are evaluated by comparing their predictions about preference in 1997, 1998, and 1999, while using the real preference during the period from 1993 to 1996 as initial status. To measure the similarity between predicting and real results, we adopt Kendall's tau coefficient [20] and the Jaccard coefficient. We individually measure the similarity for each author, each node in the network, and then average them as a performance indicator. Because Kendall's tau coefficient is not well defined with tie scores, we manually set Kendall's tau score as 0 when there is a tie on all 16 publishers. Furthermore, we calculate the Jaccard coefficient performing on the top 3 highest scored publishers.

Firstly, for the sake of knowing the correspondence between the extent of changes in iterations and in years, we execute one-iteration propagation in each model and then compare the results with the ground truth in 1997, 1998, and 1999, respectively. We also try different susceptible ratio *ϵ* in our model, as *ϵ* = 1.0 implies the authors stick to their own preferences without considering the effect from the neighbors.  Table 2 shows the results, which we find quite suitable to take one iteration as a period of a year. The results demonstrate that our model consistently outperforms the 2nd best model DiffusionRank, regardless of which susceptible ratio is used as long as it is not 1.0.

Secondly, we execute the diffusion algorithms for multiple rounds and compare them with the ground truth of years 1997–1999.  Table 3 shows the average of the scores for 1997, 1998, and 1999. Note that LT and IC models stop when there is no possible activation (regarded as one round), which implies that authors are not affected by their neighbors after the first round is completed. Tables 2 and 3 additionally show that the impervious preferences (*ϵ* = 0) reach a performance similar to the best result, which might reveal the slowly changing nature. Nevertheless, the results show that our model can faithfully capture the trait of the social influence even if the authors' interests change slowly.

## 5. A Social-Based Simulation Framework for Election Behavior

Based on the preference negotiation model, we implement a simulation framework, shown in Figure 4, granting us to analyze the social impact to elections.

### 5.1. System Architecture

Voters possess their own preference profiles to each candidate in the early stage of an election. A faithful simulation ought to produce preference profiles that are similar to the practical cases. Thus, we produce profiles satisfying certain distributions according to the data collected from a historical election.

Although we have the voter's preference data, unfortunately, there is no information telling us the relationship between the voters. To deal with this, we propose several plausible scenarios to deploy the profile on a given social network. Because people generally become friends due to similar tastes and thoughts, the profiles should not be distributed randomly. We design several plausible ways to distribute preference profiles on a social network.

Once the preference profiles are assigned to each node, the system starts executing the preference negotiation model. Nodes in the network start to persuade others and be persuaded. It is not necessary for a simulation to run until convergence, because in the real world there exist some elections which leave insufficient time for people to negotiate, debate, or exchange opinions before having to cast their votes.

**Figure 3:**
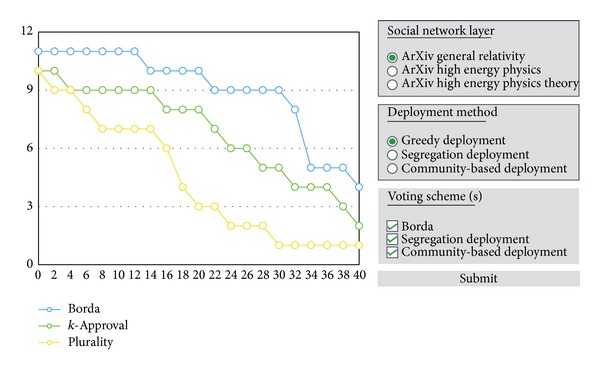
Our user-interface for the convenience of doing experiments.

**Figure 4:**
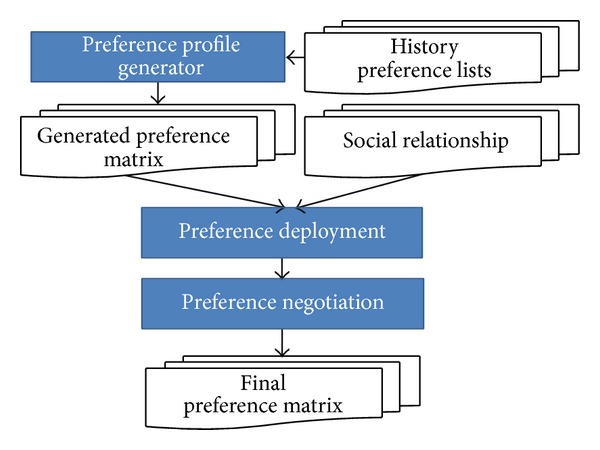
The flow chart of the proposed framework.

Finally, since in each stage there are several parameters we can adjust, we have designed a user-interface that allows the easy execution for experiments (see Figure 3).

### 5.2. Preference Profile Generation

In the Preference Profile Generation stage, we create preference profiles based on historical election data obtained from OpenSTV, an online voting record database. We choose to use the “Melbourne City Council Victoria Australia 2008—Lord Mayor Leadership team” data set because it is by far the most complete dataset we have found. This dataset was recorded in November 29, 2008, and has the ballot size of 57,961 and 11 candidates. It consists of the preference lists for all voters. A preference list is a sorted list of candidates revealing the preference order of this particular ballot.

We propose a ranking-preserved sampling method to produce the preference profiles based on the historical data, with the aim of preserving the rank of each candidate. Given our historical data, we first learn a *k* × *k* matrix *M*_*r*_, where *k* is equal to the total number of candidates. The (*i*, *j*)th elements of *M*_*r*_ encode the probability that the *j*th position in ranking belongs to candidate *i* according to the historical dataset, Prob(C_*i*_ | Rank = *j*). Each column of *M*_*r*_ yields a probability distribution for each candidate of a given rank. Given *M*_*r*_, we can iteratively sample candidates in each rank (from higher to lower) based on the distribution (with the natural restriction of prohibiting the same candidate in different positions in a single ballot).

### 5.3. Preference Deployment

As the old saying goes “birds of a feather flock together,” we presume that the people of similar preference profiles have a higher likelihood of being close to each other in the network. Below are three algorithms to realize such idea.

*Greedy Deployment*. It can be realized by first randomly picking a profile and assigning it to a node in the social network; then we assign the most similar unassigned profiles to their neighbors. Then iteratively for each unassigned node, the algorithm allocates it a profile that is the most similar to its neighbors. To measure the similarity, we exploit the commonly used Kendall *τ* coefficient:

(16) $$\begin{array}{c} \tau =\frac{\left|\text{concordant}\;\;\text{pairs}\right|-\left|\text{discordant}\;\;\text{pairs}\right|}{\left(1/2\right)n\left(n-1\right)}. \end{array}$$

*Community-Based Deployment*. Our underlying idea is to match social network communities with clusters of preference profiles. We first conduct a community detection algorithm to identify communities in a social network (in the experiment, we apply [21]), which groups the community and determines the number of communities *m* automatically based on the maximization of the modularity. Next, we apply a clustering algorithm (in this experiment, we used *k*-means) to group preference profiles into *m* clusters based on Kendall *τ* similarity.

**Figure 5:**
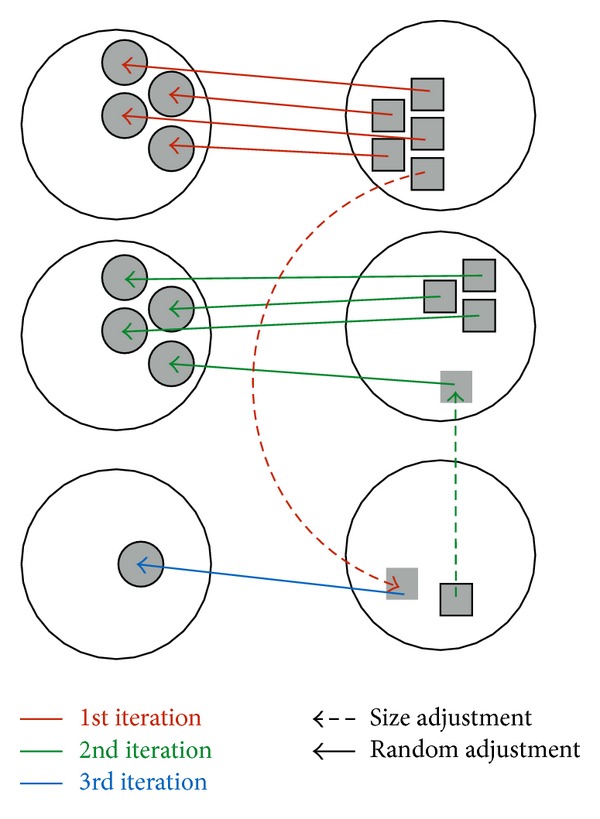
A diagram to demonstrate our deployment algorithm.

In the final step, we assign profiles to each node. The main idea is to assign each profile in the *i*th largest cluster to each node in the *i*th largest community. This, however, is not a straightforward task because the *i*th community and the corresponding cluster are likely to have different sizes. Here we propose a method to adjust the cluster sizes to match those of the communities. To accomplish this, we first sort both the sets of communities and their clusters by their respective sizes. Then from the largest community to the smallest, we compare the size of the *i*th community with that of the *i*th cluster. When the *i*th community is of the same size as the *i*th cluster, we randomly assign the profile in the cluster to a node in the community. When the cluster size is smaller, we add the unassigned points outside this cluster but closest to the cluster center into the cluster until its size matches the size of the community. When the cluster is larger, by contrast, we remove the points from the cluster that are farthest away from the center and join them to the closest unassigned neighbor cluster. Doing this iteratively will gradually assign profiles to nodes and guarantee that nodes in the same community have similar profiles. An example is illustrated in Figure 5, and pseudo code is listed in Algorithm 2.

*Segregation Deployment*. The idea is from the setting of Schelling's segregation model [22]: blacks and whites may not mind, even prefer, each other's presence, but people will move if they are the minority. At the beginning, we deploy profiles randomly. Then, in each iteration, the nodes which have fewer than 30% neighbors with positive Kendall *τ* similarity values will be selected, and their preference profiles will be shuffled. In this experiment, 1000 iterations are performed.

### 5.4. Preference Negotiation

Once profiles and communities have been assigned, the framework executes the core preference negotiation model to the network. There are two parameters that can have some impact on the process: the iterations of negotiations *R* and the susceptible ratio matrix *E*. *R* controls the negotiation iterations taken before the voters have to cast the votes. In the experiments below, we set *R* = 20.

We set *ϵ* as 0.5 for all voters by default for our case studies. As suggested in our proof, if *E* is a constant matrix *c*, then the resulting converged preference matrix *S* is indifferent no matter what *c* is (*c* only controls the speed of convergence).

## 6. Case Studies

In this section, we answer two questions based on the proposed simulation framework.

1. To what extent does the negotiation process in a social network affect election results?
2. Among the widely known voting schemas, Borda Count, *k*-approval, and plurality, which is the most vulnerable to vote-buying (i.e., easiest to be manipulated)?

In order to construct simulations, we use three collaboration networks (ca-GrQc containing 5,242 nodes and 2,890 edges, ca-HepPh containing 12,008 nodes and 237,010 edges, and ca-HepTh containing 9,877 nodes and 51,971 edges) as the underlying social network dataset. Once the negotiation process ends, the preference scoring vectors will be examined to determine the final rank of the candidates using different voting schemas: Borda Count, *k*-approval, and plurality [23]. We conduct experiments on all three plausible deployments proposed in Section 5.3. Ideally, we hope the simulation on all three deployment methods can produce similar conclusions, which would consequently offer users higher confidence about the results.

The Borda Count determines the final rank of the candidates by giving each candidate a certain number of points corresponding to the position in which it is ranked by each voter. Once all votes have been counted the candidate can be ranked by their total points. For each ballot, the top-ranked candidate will receive *k* points, the second *k* − 1 points, and so on.

The ranking in *k*-approval and plurality schemas is determined similar to Borda Count. The only difference among the three voting schemas is the definition of points to be given to each candidate. In the *k*-approval voting schema, the top *k* candidates in each ballot will each receive one point, while the rest will not receive any points. In the plurality voting schema, only the top candidate receives one point.

### 6.1. Effectiveness of Negotiation

The first question is whether the social-network-based negotiation process can significantly affect the election results. To quantify changes in a voters' preference profile through negotiations, we compute the average Kendall *τ* coefficient between the preference orders before and after each negotiation.

**Figure 6:**
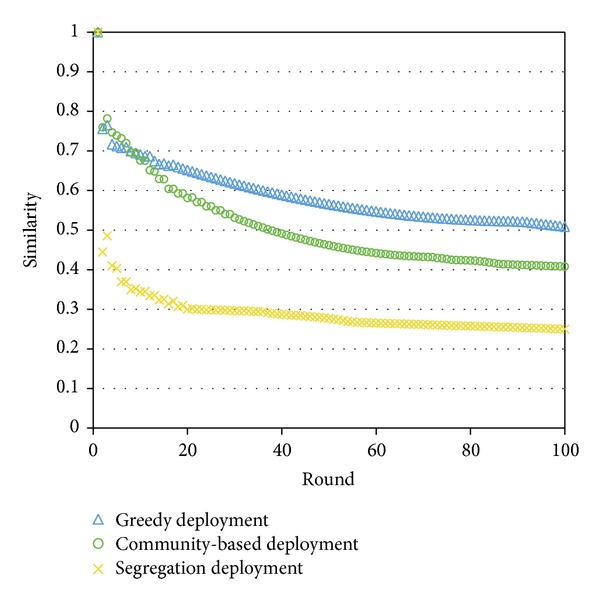
The similarity (average Kendall *τ* coefficient) between the initial preference matrix and the preference matrix after negotiations.

As shown in Figure 6, no matter which deployment method is used, the average Kendall *tau* coefficient generally decreases as we enter deeper rounds of negotiation. The slope is steepest in the beginning, revealing the fact that the effect of negotiation reaches the peak in the beginning and gradually declines, which matches the real world experience. Eventually the Kendall *tau* value decreases to below 0.5, implying that negotiation through social networks can significantly change the election results.

### 6.2. Vulnerability to Vote-Buying

This section discusses a key question about elections: if an organization can boost the vote count of a candidate through manipulating certain seed nodes' preference profiles (pejoratively, we can call this “vote-buying”). We define one successful vote-buying to a voter as “raising the target candidate's preference score to slightly higher than the score required to obtain a vote from the voter.” For example, if each voter is allowed to cast 3 votes, then the buyers would attempt raising its score to slightly above the 3rd place candidate. To quantify such manipulation cost, we defined it as the difference between the scores before and after manipulating. The scoring vector after vote-buying must be renormalized before further computation can proceed.

**Figure 7:**
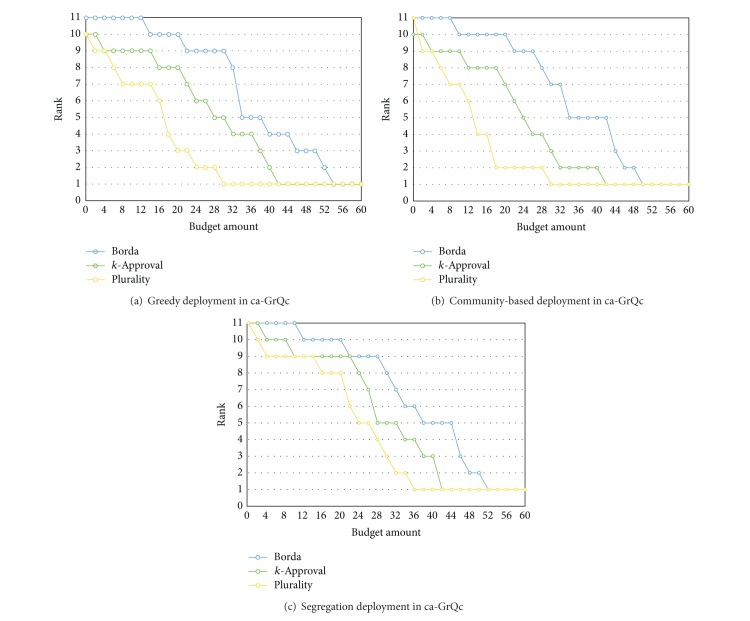
The figure shows the rank of promoted candidate in three voting schemas, where the *x*-axis stands for the budget spent while the *y*-axis stands for the rank of a given candidate for promotion. The figures in each row show the 3rd-round results with different deployments in networks ca-GrQc. The results in networks ca-HepTh and ca-HepPh are consistent. They are excluded due to the space.

Discarding the effect of negotiation, it is intuitive to assume that the best promotion strategy is aiming at the voters whose costs are the smallest. Under such attack, which voting schema is the most vulnerable to vote-buying? This answer can be deduced from Figure 7, where the *x*-axis stands for the budget spent while the *y*-axis stands for the rank of a given candidate for promotion. Note that the higher value of *y* stands for the less favorable, and a candidate has to move to lower position in order to be elected. The results show that, regardless of the deployment, Borda Count schema consistently requires more budget to advance a target candidate, while the plurality schema is the most vulnerable to vote-buying since it generally requires less budget to advance a candidate to higher rank.

## 7. Conclusion

Analyzing the effect of social networks upon group decisions outcomes is a difficult problem because it is both costly and time consuming to perform user studies to collect people's private preferences. Indeed, it is the change of preferences through social propagation in particular that we care most about, and to our knowledge this is the first ever study that provides not only theoretical analysis but also the empirical justification of this problem. This study provides an example of how to perform such research with limited data through exploiting algorithm and model design, theoretical justification, and computer simulation.

Our other significant contribution is that we provide an alternative evaluation plan and data to verify a preference propagation model. Acknowledging the lack of real world data to evaluate how the voter's preference can change through social diffusion, we have come up with a novel idea to identify a publicly available bibliography dataset to evaluate how researchers gradually change their research fields according to the influence of their collaborators. Our evaluation plan opens a new possibility that allows researchers working on preference diffusion problems to be able to evaluate their models without having to identify a highly private voter preference dataset.

## Figures and Tables

**Algorithm 1:**
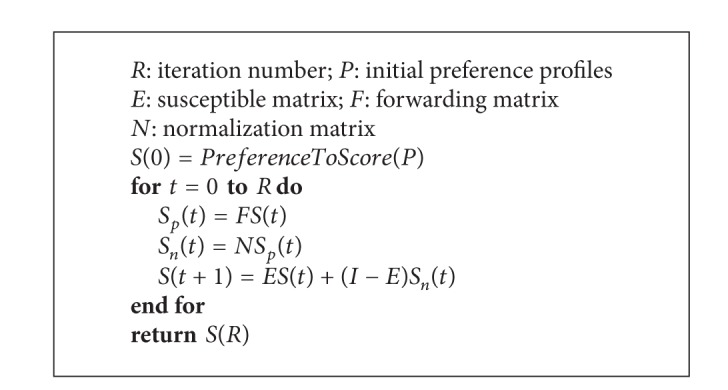
Preference propagation model.

**Algorithm 2:**
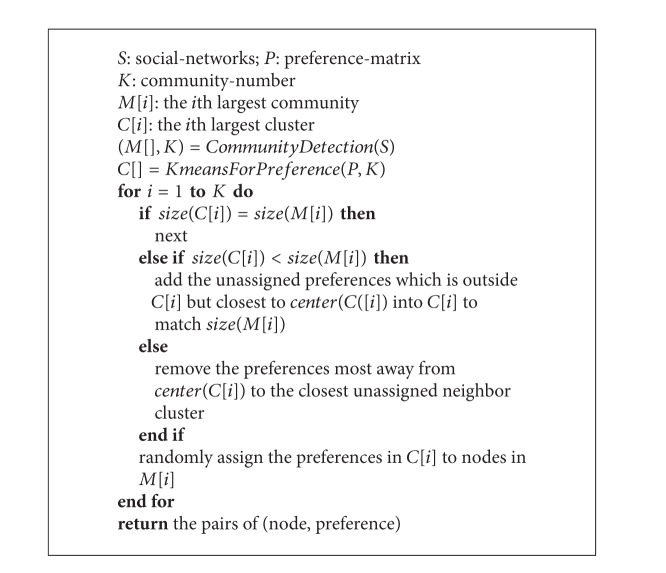
Preference deployment method.

**Table 1:** Comparison of models on the abilities to capture characteristics of real world social networks interactions.

	Convergence	Repeatability of final state	Consensus	Input dependence	Media space
Proposed model	*✓*	*✓*	*✓* (if SCC)	*✓*	*R* ^*k*^
LT model	*✓*	*✓*		*✓*	Boolean
IC model	*✓*	*✓*		*✓*	Boolean
PageRank	*✓*	*✓*			*R*
DiffusionRank	*✓*	*✓*		*✓*	*R*

**Table 2:** Compare the result after one round for each model with the ground truth of years 1997, 1998, and 1999.

	Kendall's tau			Top 3 Jaccard coefficients		
Year	1997	1998	1999	1997	1998	1999
Independent Cascade	0.007	0.012	0.015	0.011	0.014	0.015
Linear Threshold	0.172	0.167	0.167	0.171	0.195	0.212
DiffusionRank	0.221	0.181	0.160	0.216	0.222	0.213
Proposed(0.00)	0.240	0.204	0.178	0.242	0.243	0.225
Proposed(0.25)	0.243	0.206	0.180	0.248	0.244	0.226
Proposed(0.50)	0.243	0.206	0.180	0.247	0.243	0.227
Proposed(0.75)	0.243	0.206	0.180	0.246	0.243	0.226
Proposed(1.00)	0.230	0.190	0.163	0.204	0.179	0.156

**Table 3:** Consider the result after *k* × *R* rounds for each model, and compare it with the ground truth of year 1996 + *k*. The table shows the average of the similarity scores for 1997, 1998, and 1999.

	Kendall's tau					Top3 Jaccard coefficients				
Round	1	2	3	4	5	1	2	3	4	5
Independent Cascade	0.011	0.011	0.011	0.011	0.011	0.013	0.013	0.013	0.013	0.013
Linear Threshold	0.168	0.168	0.168	0.168	0.168	0.192	0.192	0.192	0.192	0.192
DiffusionRank	0.186	0.186	0.186	0.186	0.186	0.217	0.217	0.217	0.217	0.217
Proposed(0.00)	0.208	0.209	0.207	0.206	0.205	0.238	0.240	0.237	0.236	0.234
Proposed(0.25)	**0.210**	0.209	0.208	0.207	0.207	0.241	0.241	0.240	0.239	0.238
Proposed(0.50)	0.209	0.210	0.209	0.209	0.208	0.240	0.242	0.242	0.241	0.240
Proposed(0.75)	0.209	0.209	0.209	0.209	0.209	0.239	0.241	0.242	**0.242**	0.241
Proposed(1.00)	0.194	0.194	0.194	0.194	0.194	0.179	0.179	0.179	0.179	0.179

## Abbreviations

*V*: Individuals
*C*: Candidates
*n*: Number of individuals
*k*: Number of candidates
*p*_*v*_: Preference profile vector of individual *v* ∈ *V*
*s*_*v*_: Preference score vector for individual *v* ∈ *V*
*S*: Preference scoring matrix with size |*V*| × |*C*|
*G*: Social network layer.

## References

[1] Liggett T (1985). *Interacting Particle Systems*.

[2] Granovetter M (1978). Threshold models of collective be- havior. *American Journal of Sociology*.

[3] Kempe D, Kleinberg J, Tardos É Maximizing the spread of influence through a social network.

[4] Chen W, Wang Y, Yang S Efficient influence maximization in social networks.

[5] Gruhl D, Liben-Nowell D, Guha R, Tomkins A Information diffusion through blogspace.

[6] Leskovec J, Mcglohon M, Faloutsos C, Glance N, Hurst M (2006). Cascading behavior in large blog graphs: patterns and a model.

[7] Saito K, Kimura M, Ohara K, Motoda H (2010). Selecting information di usion models over social networks for behavioral analysis. *Machine Learning and Knowledge Discovery in Databases*.

[8] Ma H, Yang H, Lyu MR, King I Mining social networks using heat diffusion processes for marketing candidates selection.

[9] Yang H, King I, Lyu MR DiffusionRank: a possible penicillin for web spamming.

[10] Bartholdi JJ, Tovey CA, Trick MA (1992). How hard is it to control an election?. *Mathematical and Computer Modelling*.

[11] Gibbard A (1973). Manipulation of voting schemes: a general result. *Econometrica*.

[12] Satterthwaite MA (1975). Strategy-proofness and Arrow’s conditions: existence and correspondence theorems for voting procedures and social welfare functions. *Journal of Economic Theory*.

[13] Liu FC (2009). Modeling political individuals using the agent-based approach: a preliminary case study on po- litical experts and their limited in uence within com- munication networks. *Journal of Computers*.

[14] Yoo S, Yang Y, Lin F, Moon C Mining social networks for personalized email prioritization.

[15] Meyer CD (2000). *Matrix Analysis and Applied Linear Algebra*.

[16] DeGroot MH (1974). Reaching a consensus. *Journal of the American Statistical Association*.

[17] Winkler RL (1968). The consensus of subjective probability distributions. *Management Science*.

[18] Mossel E, Schoenebeck G Reaching consensus on social networks.

[19] Gehrke J, Ginsparg P, Kleinberg J (2003). Overview of the 2003 KDD Cup. *SIGKDD Explorations Newsletter*.

[20] Kendall MG (1938). A new measure of rank correlation. *Biometrika*.

[21] Blondel VD, Guillaume J-L, Lambiotte R, Lefebvre E (2008). Fast unfolding of communities in large networks. *Journal of Statistical Mechanics: Theory and Experiment*.

[22] Schelling T (1969). *Models of Segregation*.

[23] Johnson PE Voting systems. http://pj.freefaculty.org/Ukraine/PJ3_VotingSystemsEssay.pdf.

